# Short-Form Psychoeducation Videos: Process Development Study

**DOI:** 10.2196/66884

**Published:** 2025-07-30

**Authors:** Louise Turtle, Helen Alexandra Wesson, Simon Williamson, Nathan Hodson

**Affiliations:** 1 Warwick Medical School, University of Warwick, Gibbett Hill Road, Coventry, CV4 7AL, United Kingdom, 44 2476574880; 2 Department of Brain Sciences, Imperial College London, London, United Kingdom

**Keywords:** psychoeducation, co-production, videos, interdisciplinary development, Cognitive Behavioral Therapy, anxiety disorders, depression, psychotherapy, online therapy, mental health

## Abstract

**Background:**

Every year, around 1.8 million people in the United Kingdom are referred to NHS Talking Therapies, predominantly for cognitive behavioral therapy (CBT), which is the first-line treatment for common affective and anxiety disorders. However, more than a million of these do not complete their course. Supporting this “missing million” to attend and complete CBT is a policy priority.

**Objective:**

We aimed to coproduce a series of video resources to help patients better prepare for and complete their CBT sessions.

**Methods:**

We structured this project around a development cycle and documented outcomes against the Template for Intervention Description and Replication (TIDieR) checklist to ensure transparent intervention reporting. We assembled an interdisciplinary team to undertake an iterative video development process, composed of 3 subteams. An expert contributor subteam of 21 therapists shared their priorities and preferences for video content and style. A creative subteam of 4 members was responsible for scripting, filming, and editing video content. A project management subteam comprising 4 members (2 project managers, 1 designer, and 1 psychiatrist) distilled insights from the expert contributors and shared them with the creative team; they also presented video content to expert contributors and collected feedback. The process was terminated when expert contributors were satisfied that the videos developed could be shared with their patients.

**Results:**

We conducted 2 development cycles over 7 months between February and August 2024. In total, we produced 12 short-form videos, each 1 minute 14 seconds to 4 minutes 46 seconds long, across 4 distinct presentation styles (animation, patient narrative, therapist vignette, and expert interview). Videos covered topics such as the format of CBT (eg, why there is work to do between therapy sessions) and the psychological content (the value of developing healthy habits). Between 4 and 11 expert contributors reviewed any given batch of videos. Based on early feedback, we removed checklist formats in favor of positive storytelling, slowed pacing, and added subtitles to ensure readability and reduce cognitive load. The termination condition was achieved; expert contributors agreed to share videos with their patients.

**Conclusions:**

We successfully collaborated to produce a series of psychoeducation videos. A major strength of this process was the large number of people from different professional backgrounds involved; this diversity boosted both the validity of the content and the creativeness of the videos. This approach was well-suited to the setting of psychotherapy, where therapists have a detailed understanding of the anxieties and uncertainties of their patients, but we would advise caution in fields where professionals are less attuned to their patients’ needs. Support to engage the “missing million” is urgently needed, and psychoeducational videos provide one suitable approach.

## Introduction

### Background

In 2023, 1.8 million people were referred for NHS Talking Therapies [1]. Only 1.2 million attend their first session (the “initial assessment”), and less than 700,000 attend their second session (the first session with their allocated therapist) [1]. Improving attendance and completion of talking therapy is a key strategic goal for the National Health Service (NHS) [2]. Uncertainty and misconceptions about therapy are common barriers to attending [3], and likely contribute to the dissatisfaction that frequently underlies dropout [4].

The United Kingdom’s National Institute for Health and Social Care Research (NICE) recommends cognitive behavioural therapy (CBT) as first line for mild and moderate depression, with or without accompanying antidepressant psychopharmacological therapy [5]. NICE also recommends CBT and related therapies as first-line treatment for anxiety disorders, such as generalized anxiety disorder and social phobia, again, with or without antidepressant psychopharmacological therapy [6]. In the United Kingdom, primary care doctors (known as general practitioners) commonly commence antidepressant medication, but historically referred less than 5% of patients with anxiety and depression to an evidence-based psychological treatment [7]. In response, the UK government created the initiative *Improving Access to Psychological Therapies*, now known as “NHS Talking Therapies,” in 2008. This initiative increased therapy uptake by offering people in the United Kingdom with depression or anxiety disorders the opportunity to self-refer for a course of talking therapy, typically lasting 8 sessions, while also increasing the therapy workforce by training more than 10,000 new therapists over 10 years [8]. People who self-referred would receive an initial evaluation to appraise their suitability for talking therapy and to establish what form of talking therapy would be most suitable. NHS Talking Therapies has proven an effective approach. Meta-analytic evidence indicates large reductions in depression and anxiety and a medium-sized improvement in work and social adjustment [2].

While demand for NHS Talking Therapies support has increased over the intervening period, engagement has remained inconsistent; so much so that the program has been described as “hemorrhaging patients” [9]. In 2013‐2014, there were 1.3 million referrals into *Improving Access to Psychological Therapies*; 42% (468,881/1,267,193) completed sufficient sessions [10]. In 2023‐2024, 37% (670,000) of the 1.8 million people referred actually completed therapy [1]. Unfortunately, there is also no evidence that the “missing million” are either fully recovered or unsuitable for therapy. Therefore, there is a clear need to improve engagement among this group.

High rates of attrition from psychotherapy are widely acknowledged and present a significant challenge for evidence-based psychotherapy services [1,2,11]. In 2010, Self et al [3] drew on data from the United Kingdom NHS to argue that the only demographic factor consistently correlated with poor attrition in the literature is socioeconomic status, leaving extensive scope for individual differences based on beliefs about mental illness and therapy. Since then, there has also been a clear divergence of Pakistani and Bangladeshi British people whose engagement in NHS Talking Therapies has been lower than the median, and this may also be partially attributable to differences in beliefs about mental illness and therapy among other factors [1,12]. Globally, the picture is different, and international meta-analyses drawing heavily on other health care systems have found lower rates of discontinuation (ranging from 20% to 47%) [13,14]. Minority racial status has been associated with dropout in a predominantly North American meta-analysis, although socioeconomic status could not be included due to lack of data [13]. In another North American meta-analysis, race was not a significant predictor of engagement [14]. Diagnosis of a personality disorder or treatment in a university predicted lower engagement, but it is not relevant to NHS Talking Therapies, which focuses on uncomplicated anxiety and depression treated in local NHS clinics. These findings demonstrate, first, the value of considering pathways in isolation, rather than pooling data across health systems [3]. Second, the lack of a clear picture of demographic drivers of attrition may indicate that individual differences in health beliefs explain why some people attend and others do not [3].

One approach to long CBT waiting lists is fully remote, asynchronous CBT. The potential advantages include the convenience of therapy in your own home at your own pace [15]. These approaches, with or without light-touch human support, have had some success, but engagement with fully remote asynchronous programs remains as low as 3% to 25%, partially due to the burdensome and unexciting nature of the content, and patients’ wariness of digital therapy [15-17]. Engagement challenges in remote asynchronous CBT have become so significant that Batterham et al [18] developed a digital intervention to increase engagement with their digital intervention. While the long-term solution may be fully remote asynchronous therapy, over the medium term, other means of increasing engagement with human therapists are needed.

In 1992, Hunt and Andrews [19] argued that “the finding that dropouts are ubiquitous in psychotherapy is very damaging, for if patients do not stay for treatment, then there is little point in developing effective treatment” and in 1998, Harris [20] made this call to action: “it is necessary for investigators to move beyond research on correlations of attrition to propose and test theoretical models with clearer implications for preventing attrition.” Over the subsequent 27 years, the knotty problem of attrition from psychotherapy remains inadequately addressed, but meta-analysis data shows that increasing the credibility of CBT leads to improved treatment outcomes [21]. In this paper, we worked with NHS Talking Therapies professionals to gather their understanding about why people drop out before or during therapy and with a creative team to develop a psychoeducation intervention to address these concerns.

### Aims

This study aimed to develop a series of video resources to reduce nonadherence by providing accurate and trustworthy information about NHS Talking Therapy in an engaging manner.

## Methods

We assembled an interdisciplinary team and undertook an iterative process of video development [22-24]. All development work took place over a 6-month period between February and August 2024.

### Content Team Composition

**Figure 1.**
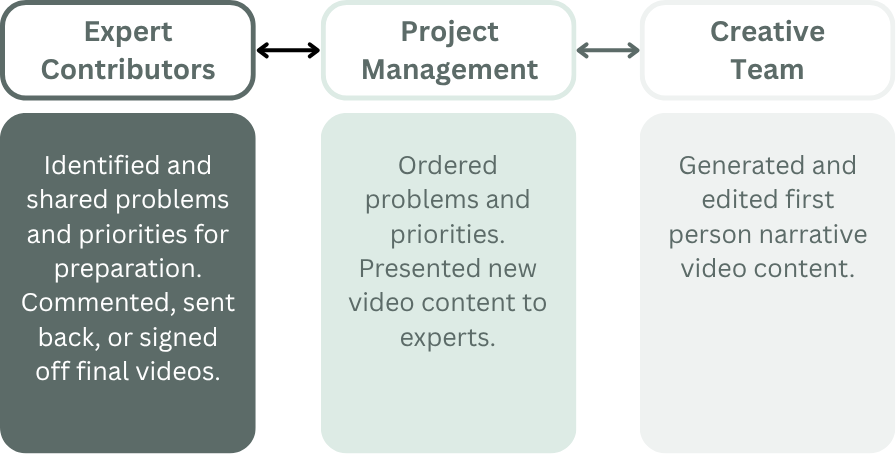
The 3 subteams.

Building on the methodology reported by Rosaasen et al [23], we assembled a team with the appropriate set of skills to address the development challenges. A project management subteam facilitated collaboration between a creative subteam (1 psychiatrist, 2 actors, and 1 videography expert) and a subteam of NHS Talking Therapies expert contributors. The project management team liaised with the expert contributors to establish their content ideas and proposed changes, and to liaise with the creative team to present feedback and discuss content development, as illustrated in Figure 1. Although patients were not included in any of the 3 teams, preparatory work was conducted during early 2024 with a range of stakeholders, including 3 patient groups and 2 consultant experts-by-experience, as this creative process was being developed.

The expert contributors and project management team conducted frequent meetings over the course of the 6-month project. The project management team collated the insights of the expert contributors and presented these to the creative team. The creative team generated multimedia insights and shared them with the project management team, who in turn sought feedback from the expert contributors.

The project management team, recruited through the University of Warwick, had previous expertise in digital project development. It comprised 2 project managers, 1 designer, and 1 psychiatrist. The expert contributors were recruited directly and through snowballing on the basis that they were involved in the provision of CBT services; they included 16 clinicians from NHS mental health trust A and 5 from NHS mental health trust B. A total of 11 of these subject matter experts made repeated contributions throughout the feedback process. The creative team was identified through participation in medical school or community creative projects and included 5 people (3 actors, 1 animator, and 1 psychiatrist).

### Content Development Cycle

Video presentations of the psychotherapy experience have previously been identified as an effective way of communicating relevant information, as they have in other contexts [25]. In stage 1a, the project management team established the priorities and preferences of the expert contributors. This was done through a series of meetings between the 2 subteams. Minutes were taken by HAW at each meeting and integrated with information gathered in previous meetings.

In stage 1b, these minutes were presented to the creative team. Within the creative team, the core problem or priority from the expert contributors was distilled, and a narrative was developed. Then, 1 or 2 members of the creative team were allocated the task of writing a short script. This script was edited by other members of the creative team and the project management team. Once the script was agreed, 1 member of the creative team was allocated to make the video through live action filming or animation. The final product was edited using VEED.IO, video production platform (VEED Ltd), followed by final touches (including subtitling).

In stage 2a, the videos were presented to the project management team with any necessary context. The project management team presented the videos back to the expert contributors for their comments. Any further comments from the expert contributors were recorded and delivered back to the creative team with the next set of priorities and preferences. In stage 2b, any further changes required were made either in VEED.IO or through rerecording, and the video was exported and returned to the expert contributors.

This cycle of development and feedback continued for each video until the expert contributors reported that they were happy to share the video with potential users. Given the busy schedules of the expert contributors, most videos were seen by more than 1 expert contributor; any 1 expert contributor could “sign off” on a video or send it back. This process resulted in a series of videos that could be offered to potential service users by expert contributors [26].

### Describing the Intervention

The resulting psychoeducation intervention was produced and described using the Template for Intervention Description and Replication (TIDieR) checklist [27]. The TIDieR checklist provides a consistent framework for researchers to describe interventions.

### Ethical Considerations

The Biomedical Sciences Research Ethics Committee at the University of Warwick confirmed that this project was exempt from human subject research ethics review as there were no human research subjects. In the absence of human subjects, informed consent was not relevant, nor was confidentiality or compensation.

## Results

### Timeline of Events

Table 1 illustrates a timeline of the development events.

**Table 1.** Timeline of events.

Month	Stage	Subject matter expert progress		Video progress
		Subject matter experts	Meetings	
June	Stage 1	11	2	—^a^
July	Stage 2‐3	9	5	Initial content production
August	Stage 4‐5	10	8	Updates from creative team
September	Stage 5	4	2	Termination criteria reached and intervention shared

a Not applicable.

### Stage 1: Preferences and Priorities Identified by Expert Contributors

The contributing experts reported a wide range of priorities to the project management team. Their recommendations included topics relating to explaining the format of CBT, describing some psychological theory, and using an accessible design style. With respect to the CBT format, the expert contributors suggested describing some features that are uniform to CBT and others that are optional, as well as considering practical considerations for sessions. With respect to the more psychological content, they advised describing the value of building healthy habits and relaxation skills before therapy, the importance of safety from deliberate self-harm and suicide, and the importance of participation in recovery. Finally, they suggested that the style should reflect modern video provision on social media, such as short-form videos, and should draw on color schemes that are suitable for people with neurodevelopmental differences.

### Stage 2: Initial Content Production

The creative team scripted, filmed, and edited a pilot series of 8 videos illustrating these messages and including “checklists” of steps to take before beginning CBT. Topics covered were as follows: (1) What is CBT? (2) Where did CBT come from? (3) What can CBT help with? (4) How I told my boss about CBT? (5) How I told my children about CBT? (6) How I told my partner about CBT? (7) A day in the life of a CBT therapist. (8) There is homework in CBT? Furthermore, 4 styles were developed: a corporate information animation (videos 1-3), a friendly former patient (videos 4-6), a therapist (video 7), and an expert interview (video 8). Videos ranged from 1 minute 14 seconds to 4 minutes 46 seconds.

### Stage 3: Improvements Suggested by Expert Contributors

The expert contributors rereviewed the initial content developed. They were generally content that the information given about the content of CBT was accurate. However, they were concerned that a proposed checklist of things to do before CBT could be overwhelming for service users. Expert contributors identified several technical points on which the videos could be improved. With respect to the breadth of conditions treated with CBT, they suggested that mentioning dementia could be confusing for service users, but thought that the potential for CBT to help with chronic health conditions should be discussed. They also advised against using the concept of misconceptions and instead focused on facts and positive stories of CBT’s success. Many additional comments related to style were made; they felt initial videos presented too much information too fast, and that key content should be in written form too, not just audio.

### Stage 4: Updates Made by Creative Team

The creative team responded to all of the above comments. The concept of checklists was removed completely to remove the binary implication that some people are “ready” and others are “not ready” for CBT. Named conditions were changed, and the focus on anxiety and mood disorders was emphasized, while chronic physical health conditions were included. Likewise, the idea of misconceptions was removed and replaced with more positive framing. Stories of CBT were made more prominent by developing further scripts from the perspective of the friendly former patient. Videos were slowed down, scripts were shortened, and subtitles were added.

Table 2 describes the development process from stage 1 to stage 4.

**Table 2.** Summarizing stage 1 to stage 4.

	Stage 1: Examples of key preferences and priorities	Stage 2: Which content covered it?	Stage 3: Suggested improvements	Stage 4: Changes made by the creative team
Information on CBT^a^ format				
	There is homework in CBT. Therapists are trained professionals.	7, 8	Checklists to describe practical steps are overwhelming	Checklists were removed.
	Tell the therapist about any personal preferences.	7, 8	—^b^	—
	Consider practicalities such as where CBT happens, transport to CBT during the working day, and balancing CBT with other commitments.	4, 5, 6	—	—
Information about psychological theory				
	Develop healthy habits in advance such as relaxation activities and sleep.	1, 3, 7	Remove dementia, mention chronic conditions.	Main conditions changed.
	Psychological concepts such as thoughts-body-actions-physical feelings.	1, 2, 3, 8	Add stories of CBT benefits and consider mentioning safety on the waiting list	—
	Recovery is not immediate or not guaranteed. Recovery is more likely with CBT.	3, 5, 6	Avoid the concept of “misconceptions”	The concept of misconceptions is replaced with positive framing and positive stories.
Stylistic suggestions				
	Vary audio-visual style and include colors to suit neurodevelopmental differences.	1, 2, 3, 4, 5, 6, 7, 8	Too fast, keep information manageable, make it readable for people who prefer to read	Scripts shortened, subtitles added.

a CBT: cognitive behavioral therapy.

b Not applicable.

### Stage 5: Resulting Intervention Presented to Expert Contributors

After the stage 4 changes were made, the project management team presented the resulting intervention to the expert contributors. The expert contributors were happy to share the videos with their users, and the cycle was terminated. Table 3 describes the resulting intervention, a series of short-form videos, using the TIDieR format [27].

**Table 3.** Describing the digital intervention.

TIDieR^a^ criteria	Component
Brief name	Get ready for therapy video series
Why	After referral, there are low rates of initiation and completion of CBT^b^ through NHS^c^ Talking Therapies. Setting expectations and informing prospective patients about CBT is an appropriate first step in addressing this.
What	A short-form video series was developed and no additional interactions were required. Exemplar materials can be accessed online [28].
Who provided	Requires an intervention assistant to share sequential videos via WhatsApp (Meta) or email.
How	Internet, asynchronous, individual.
Where	Internet connection required, access to WhatsApp or email either via smartphone, tablet, or computer.
When and how much	12× short form videos each lasting 1‐5 minutes. Order given: Watched all at once, or one by one, with the option to rewatch videos later.
Tailoring	Tailoring not required, just targeted for people awaiting NHS Talking Therapies.
Modifications	—^d^
How well (planned)	—
How well (actual)	—

a TIDier: Template for Intervention Description and Replication.

b CBT: cognitive behavioral therapy.

c NHS: National Health Services.

d Not applicable.

## Discussion

### Summary of Findings

The aim of this study was to develop a series of videos to help people prepare for CBT within the NHS Talking Therapies service. Between February and August 2024, we conducted an iterative process of development, combining 21 expert contributors, 4 members of the project management team, and 4 members of the creative team. We produced 12 short-form videos lasting 1-5 minutes. The videos used 4 different styles and covered information about the format of CBT as well as psychological theory. By the end of the second cycle of development, partners at NHS trusts were satisfied by the quality and content and shared videos with patients, achieving the termination condition. We concluded that an iterative interdisciplinary approach is a feasible approach to coproducing psychoeducational videos to the standard required by NHS psychotherapists.

### Comparison With the Literature

#### Approaches to Increasing Engagement With Therapy

Web-based interventions similar to these videos have been designed to increase engagement with eating disorders services. Muir et al [29] developed the MotivATE website to improve engagement with NHS eating disorders services and drew on patient stories. McLean et al [30] developed the Reach Out and Recover website to improve engagement with Australian Eating Disorders services. One advantage in the case of NHS Talking Therapies waiting lists is that patients have already taken the step to seek help, although the long waiting list creates a risk of disengagement. In keeping with those interventions, we anticipate that the use of patient stories of success will reduce levels of disengagement.

#### Generalizability of the Content Produced

This approach has produced several videos which are highly specific and targeted to the context of NHS Talking Therapies service. While NHS Talking Therapies is by far the largest route to psychotherapy in the United Kingdom, other routes include private providers, university well-being services, and specialist mental health teams [31]. The videos produced in this project are of mixed relevance to these other routes into therapy. Likewise, some would be partially appropriate for those accessing health care systems in other countries, but not all. Videos addressing the core mechanisms of CBT would be applicable widely. However, several “confessional” style videos specifically mentioned a referral from a general practitioner (primary care physician) which could be distracting for those accessing psychotherapy through other routes and could be confusing in many countries where primary care is arranged differently.

### Strengths and Limitations

This is the first study to describe the process of developing psychoeducation videos. A major strength is the large number of professionals involved, which boosted both the validity of the content and the creativity of the videos. The use of subteams (expert contributors, project management, and creative) ensured that we benefited from individuals’ areas of expertise, without expecting people to work outside their areas of expertise (eg, asking a psychotherapist to script a video or asking an actor to research the details of therapy). However, the team composition also included a psychiatrist and an actor who is a CBT trainee in the creative team; their presence enabled rapid factual clarification rather than relying entirely on the development cycle.

Another strength arose from the commitment of the expert contributors. The novel concept and high-quality production of the initial videos built enthusiasm among expert contributors who were invested in reviewing the products of the second cycle when they saw the initial videos addressing their preferences and priorities.

The primary limitation of the study is that we did not include anybody currently on the waiting list in the development process. Some would argue that psychoeducation videos should be developed with people who are on the waiting list or were on the waiting list in the past. Yet, many other interventions designed to improve engagement with therapy have also been developed by expert panels without direct patient input [32,33]. In our case, each of the expert contributors had seen tens or hundreds of patients, so drawing on the expert opinion of therapists acted as an impact multiplier. There may be some features of the therapy experience which patients hide from therapists, yet demographically it is highly likely that several expert contributors have lived experience as psychotherapy patients (as colleagues, it would have been inappropriate to ask about this directly). Furthermore, 2 of the paper authors (HAW and NH) are disclosed former psychotherapy patients, further addressing this limitation. Finally, to be sure, former patients turned mental health professionals are likely a more engaged subset of patients than those who do not pursue careers in related fields, but any subgroup of patients willing to spend time in coproduction would also be self-selecting and more enthusiastic than the average participant. By contrast, the professional opinion of our expert contributors was derived from experience treating patients across the entire spectrum of people attending CBT. Nevertheless, we acknowledge that some patients fail to attend even an initial assessment appointment, and therefore, the therapists included do not necessarily have an insight into their mindset beyond what they read in the initial referral. This population is inherently difficult to engage, but in further research, we aim to include them as participants.

### Implications for Research and Policy

In general, we cautiously commend this iterative interdisciplinary method of video development to other researchers. This project succeeded in producing videos that therapists considered valuable, illustrating the value of employing a nodal project management team to coordinate a creative team and an expert contributor team. However, our approach relied on the deep understanding of expert contributors regarding the preferences and feelings of their patients; we cannot guarantee that professionals who are not psychotherapists will have the same depth of insight into patients’ thoughts, so this approach should be used with caution in other fields. In addition, it is as yet unclear whether these videos are in fact beneficial for people on the waiting list; further study is required to investigate this.

Further research should formally evaluate whether these videos are acceptable and engaging for patients. Then, the objective effects of the intervention on patient engagement and outcomes of psychotherapy should be assessed. Standardized measures of engagement and outcomes are used by the national NHS Talking Therapies program, but qualitative interviewing is more likely to be suitable for evaluating the effect of the videos of subsequent experience of psychotherapy [11]. Randomized controlled trial evidence is important in this field because behavior change interventions in this field have previously led to counterintuitive results [18,34]. If further evidence supports the usefulness of these videos, they could be disseminated through a website or app to reach a wide number of patients in the NHS and other health care systems in a highly scalable way.

Finally, this study illustrates the feasibility of rapidly and cheaply developing psychoeducation materials in a format readily accessible to contemporary tastes. The continued use of paper leaflets and text-dense websites is increasingly difficult to justify; indeed, the validity of *informed* consent could be called into question if patient information materials fail to keep up with the progressing expectations and preferences of patients [35]. It is incumbent upon health care professionals to produce information materials in a convenient and engaging format for patients, and this project has demonstrated that it is possible to do so efficiently and effectively.

### Conclusions

We developed a video series to help people prepare for NHS Talking Therapies CBT through an iterative interdisciplinary process. Our experience highlights the value in collaboration between mental health professionals and creatives in helping the “missing million” make the most of their opportunity to benefit from CBT through the NHS.

## Abbreviations

CBT: cognitive behavioral therapy
NHS: National Health Service
NICE: National Institute for Health and Social Care Research
TIDieR: Template for Intervention Description and Replication
